# Supplementary material for: A Simplified Method for CRISPR-Cas9 Engineering of Bacillus subtilis
Source: Microbiol Spectr. 2021 Sep 15;9(2):e00754-21. doi: 10.1128/Spectrum.00754-21 (PMC8557940; doi:10.1128/Spectrum.00754-21)
Supplement: SUPPLEMENTAL FILE 1 — Supplemental material. Download SPECTRUM00754-21_Supp_1_seq5.pdf, PDF file, 1.2 MB [file spectrum00754-21_supp_1_seq5.pdf]

SUPPLEMENTARY INFORMATION

A simplified method for CRISPR-Cas9 engineering of *Bacillus subtilis*

Ankita J. Sachla, Alexander J. Alfonso, John D. Helmann

Table S1: Strains used in the study.

Table S2: Oligos used in the study.

Fig S1: Detailed outline of CRISPR-Cas based editing strategy.

Fig S2: General strategy for assembly of repair template.

Fig S3: pAJS23 plasmid map and sequence (text) file

Table S1. Strains used in the study

Strain	Genotype	Construction	Reference
E. coli			
ECE358	*pJOE8999 (kan)*		(1)
HEAS25630	*pJOE8999-gRNA(erm)*	pAJS23→DH5α	This study
HEAS25655	*pJOE8999-gRNA(erm)-prkC-Sau-rpe(repair)*	pAJS24→ DH5α	This study
HEAS25666	*pJOE8999-gRNA(erm)-prkC-Sau-rpe(repair)*	pAJS24 → TG1	This study
HEAS1215	p*JOE8999-gRNA*(*erm*)*-yqgC-sodA* without S936 (Ref nt. position 2586043-2586220 deleted)-repair template	pAJS28→DH5α	This study
HEAS1223	p*JOE8999-gRNA*(*erm*)-yqgC-sodA without S936 (Ref nt. position 2586043-2586220 deleted)-repair template	pAJS28→TG1	This study
HEAS1262	p*JOE8999-gRNA*(*erm*)*-yceF**-Ile206Thr(repair)	pAJS27→DH5α	This study
HEAS1258	p*JOE8999-gRNA*(*erm*)*-yceF**-Ile206Thr(repair)	pAJS27→TG1	This study
HEAS1183	p*JOE8999-gRNA*(*erm*)*-yqgB-yqgC-gfp-sodA*(repair)	pAJS26→DH5α	This study
HEAS1185	p*JOE8999-gRNA*(*erm*)*-yqgB-yqgC-gfp-sodA*(repair)	pAJS26→TG1	This study
HEAS1214	p*JOE8999-gRNA*(*erm*)*-yqgB-yqgC-sodA-FLAG-yqgE* (repair)	pAJS25→DH5α	This study
HEAS1222	p*JOE8999-gRNA*(*erm*)*-yqgB-yqgC-sodA-FLAG-yqgE* (repair)	pAJS25→TG1	This study
HEAS1206	p*JOE8999-gRNA*(*erm*)*-yqgB*-scar-*sodA -yqgE* (repair)-0.5kb length	pAJS29→ DH5α	This study
HEAS1212	p*JOE8999-gRNA*(*erm*)*-yqgB*-scar-*sodA -yqgE* (repair))-0.5kb length	pAJS29→TG1	This study
HEAS1206.1	p*JOE8999-gRNA*(*erm*)*-yqgB*-scar-*sodA -yqgE* (repair)-1.5kb length	pAJS30→ DH5α	This study
HEAS1212.1	p*JOE8999-gRNA*(*erm*)*-yqgB*-scar-*sodA -yqgE* (repair))-1.5kb length	pAJS30→TG1	This study
B. subtilis			
168	*trpC2*	Lab strain	Lab stock
CU1065	*trpC2 att SPβ* (WT)	Lab strain	Lab stock
HB20401	*trpC2 cpgA*::*erm*	BGSC	Lab stock
HBYL844	*trpC2 cpgA*::*cpgA-Sau*	pAJS24$_{CRISPR}$→HB20401	This study
HBYL264	*trpC2 yqgC::erm*	*BGSC yqgC::erm* → CU1065	This study
HBYL1239	*trpC2* Δ2586043-2586220 intergenic deletion between *yqgC-sodA*	pAJS25$_{CRISPR}$→ HBYL264	This study
HBYL344	*trpC2 yceF*::*erm*	BGSC yceF::erm --> 168	Lab stock
HBYL1260	*trpC2 yceF* (Ile206Thr)*	pAJS26$_{CRISPR}$→ HBYL344	This study
HBYL1246	*trpC2 yqgC-gfp*	pAJS27$_{CRISPR}$→ HBYL264	This study
HBYL1249	*trpC2 sodA-FLAG*	pAJS28$_{CRISPR}$→ HBYL264	This study
HBYL260	*trpC2* Δ*yqgC*	PAJS29 $_{CRISPR}$→ HBYL264	This study
HBYL261	*trpC2* Δ*yqgC*	PAJS30 $_{CRISPR}$→ HBYL264	This study

Table S2. Oligos used in the study

Primer Name	Sequence	Reference
ermgRNAF	TACGTTTGAAATCGGCTCAGGAAA	This study
ermgRNAR	AAACTTTCCTGAGCCGATTTCAAA	This study
prkCFrepair	**AAGGCCAACGAGGCC**ACGGATCCTAAAGCGGATACCACAG	This study
LFHprkC-Sau-rsgA-R	GATTTCACTATTCGACCTGTCTTCAAATTTTCCCTCCTTGTTATTCATCTTTC	This study
LFHprkC-Sau-rsgA-F	GAAAGATGAATAACAAGGAGGGAAAATTTGAAGACAGGTCGAATAGTGAAATC	This study
Rpe-downrepair-R	**AAGGCCTTATTGGCC**TAGATCGGGAATGAGATTTTTCGGGCCTC	This study
yqgC1-seqF	CTTTTGTCATTCCGGTTGCAGGGATC	This study
gfp1-seqR	AAGTTTTCCGTATGTTGCATCACCTTCAC	This study
0.5kb-yqgC-F	**AAGGCCAACGAGGCC**CCCTGTACTTACATAATAAG	This study
0.5kb-yqgC-R	TTGGCCAATAAGGCCTCACGTATGTGTTGTGGTGTTTC	This study
0.725kb-yqgC-up-F	**AAGGCCAACGAGGCC**AATAAGCAGAAAGCTTCCAGAGCTG	This study
GFP-LFHF	ATGATCGGTTACTTTTTATGGACGGTCCTACGTAAAGGAGAAGAACTTTTCACTGGAGTT	This study
GFP-LFHR	CATTAAACCTGCCGCCAGCATTTCTTATTTGTATAGTTCATCCATGCCATGTGTAATC	This study
1.463kb-yqgC-R	**AAGGCCTTATTGGCC**CACGACAGCCAGGGCAAATAAACTG	This study
1.028kb-yqgC-F	**AAGGCCAACGAGGCC**TATTGTCCAGAGCTTGTTC	This study
YqgC-NOS936-LFHF	GTTACTTTTTATGGACGGTCCTATAAATGGCTTACGAACTTCCAGAATTACCTTATG	This study
YqgC-NOS936-LFHR	CATAAGGTAATTCTGGAAGTTCGTAAGCCATTTATAGGACCGTCCATAAAAAGTAAC	This study
0.691-yqgC-R	**AAGGCCTTATTGGCC**TAGTCTGTCTCACTCAAAC	This study
sodA-FLAG-LFHF	CAAAAGATTATAAAGATGATGATGATAAATAA TGGCACAAACAAGGTCCTCATTATG	This study
sodA-FLAG-LFHR	TTATTTATCATCATCATCTTTATAATCTTTTGCTTCGCTGTATAGACGAGCCACTTC	This study
1.571-sodA-R	**AAGGCCTTATTGGCC**ATCGGAAGCGACTTCTTTTTCTCTTTATG	This study
yqgC-seqF	GAGGCAGTTTTTGCCGCAGTATTATTC	This study
sodA-seqR	TGTCCAGAATAATTTGTGGTTCGCGTG	This study
yceE-repair-F	**AAGGCCAACGAGGCC**CAGTATGGCCATTCAATTATCAAAAGG	This study
yceG-repair-R	**AAGGCCTTATTGGCC**AACCGAGCTCCTGCTTCGGGTCGAC	This study
yceF-206-LFH-Fw	CACAGCATTCGTCTTAATCGGCATTACCGCGCTGAAAATGGCGGGGAGCGCTTTC	This study
yceF-206-LFH-Rev	GAAAGCGCTCCCCGCCATTTTCAGCGCGGTAATGCCGATTAAGACGAATGCTGTG	This study
yceF-seqF	GCAAAAGTGTTCCTTGTGCTGATTGA	This study
yceF-seqR	GTTTTATTCTTCTTTTGAAGCGGCTG	This study

Sequences shown in red or blue are recognition sites for SfiI used for cloning of repair templates into pAJS23.

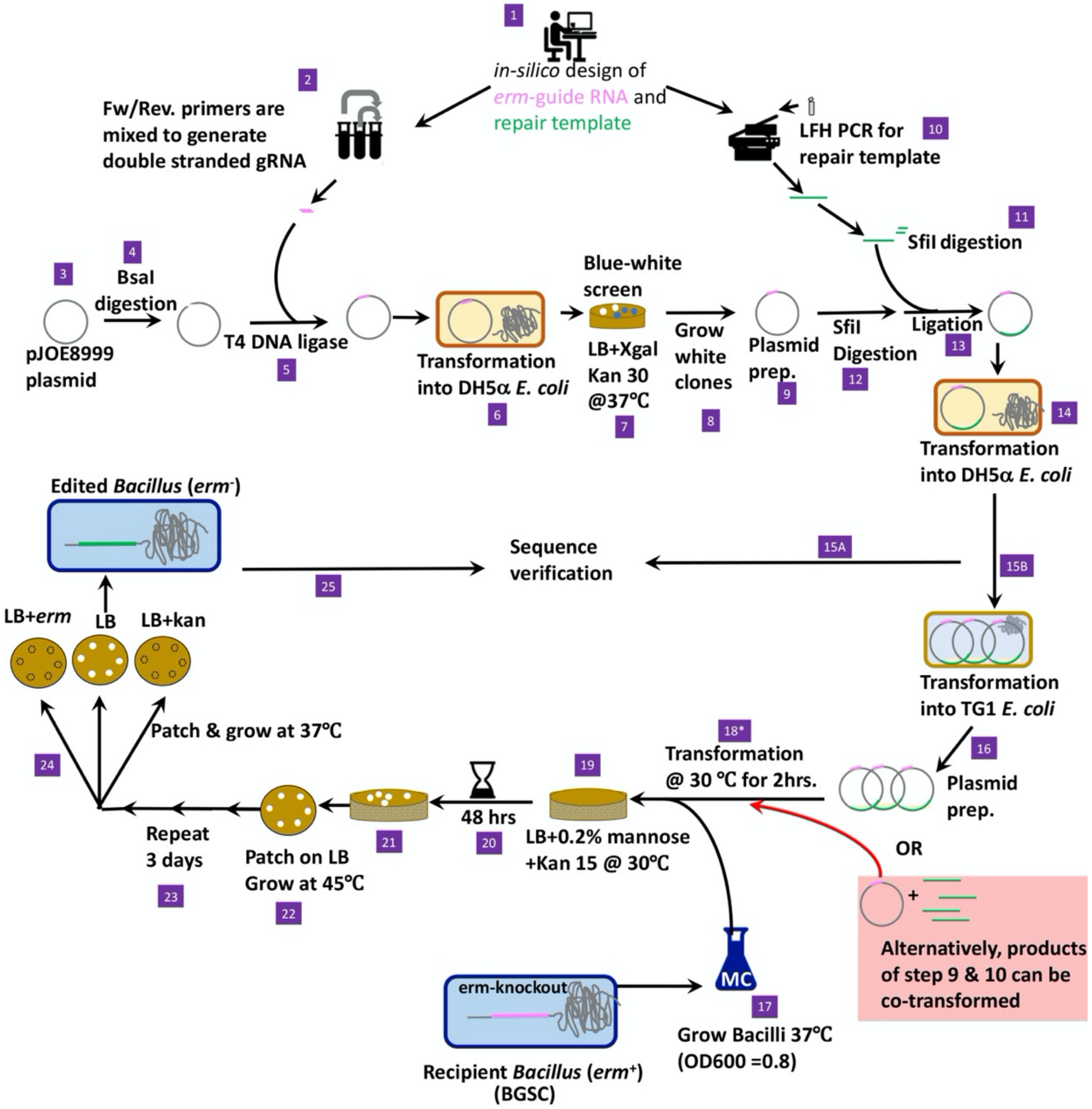

Fig S1: **Detailed outline of CRISPR-Cas based editing strategy.** Steps 1-8 shows *erm*-guide RNA cloning in pJOE8999 (ref. 1) to generate pAJS23 (step 9). For the applications here, begin at step 9 and clone a repair template into pAJS23 at the SfiI sites, or instead provide a suitable repair template in the form of either a PCR product or genomic DNA as shown in pink box at step 18* with red arrow. A detailed protocol follows:

For genome-editing with a repair template cloned into pAJS23

Design a suitable repair template (step 1) by, for example, amplification by PCR using primers that include SfiI restriction enzyme sites (step 10). The repair template is cleaved with SfiI (step 11) and ligated (step 13) into pAJS23 (from step 9) that has been digested with SfiI (step 12). Ligation is catalyzed by T4 DNA ligase at room temperature for 1 hr (or 18 hours at 16 °C). Optional: ligation reaction mixture could be heat inactivated at 65 °C for 20 minutes. Next,

transform *E. coli* DH5α (step 14) with selection for kanamycin (30 μg ml^{-1}). The correct plasmid should be verified using analytical PCR (step 15A). The desired plasmid is then transformed into *E. coli* TG1 *E. coli* (generates multimeric plasmid DNA; step 15B) and then cultured to prepare a plasmid miniprep (step 16).

For genome editing, an appropriate recipient strain (any strain containing an integrated *erm* cassette at the locus targeted for modification, including *Bacillus subtilis* 168 strains from the BKE collection) is transformed with the editing plasmid (step 18*). 1 ml of the transformation reaction is plated on LB plates containing 0.2% mannose (to induce gRNA expression) and 15 μg ml^{-1} of kanamycin (to select for plasmid in *B. subtilis*) at 30 °C for 48 hours (step 20). Also plate a negative control with no input DNA.

Patch or re-streak *B. subtilis* clones from the LB kanamycin plates onto LB plates with no antibiotics at 45 °C for 18 hours to cure the plasmid (step 22). Repeat this process for 2 to 3 consecutive days (step 23). The efficiency of curing is best if colonies are highly diluted during patching or streaked to single colonies. Test isolated candidate colonies for antibiotic sensitivity by streaking on LB plates plus MLS (1 μg ml^{-1} of erythromycin, 25 μg ml^{-1} of lincomycin) and LB plus 15 μg ml^{-1} of kanamycin (step 24). Clones that show no growth on kanamycin are presumed to have lost plasmid. MLS^S clones have lost (or inactivated) the *erm* cassette of the targeted gene. Test candidate recombinants by colony PCR and DNA sequencing to ascertain introduction of desired genomic edit (step 25).

For genome editing with pAJS23 and a co-transformed repair template

Proceed as above, using 1 μg pAJS23 (from step 9) and the desired repair template at step 18*. Repair templates can be prepared by sequence overlap extension (SOEing) PCR or by preparing genomic DNA from a donor strain containing the desired mutation or genetic construction. Include a control with no repair template.

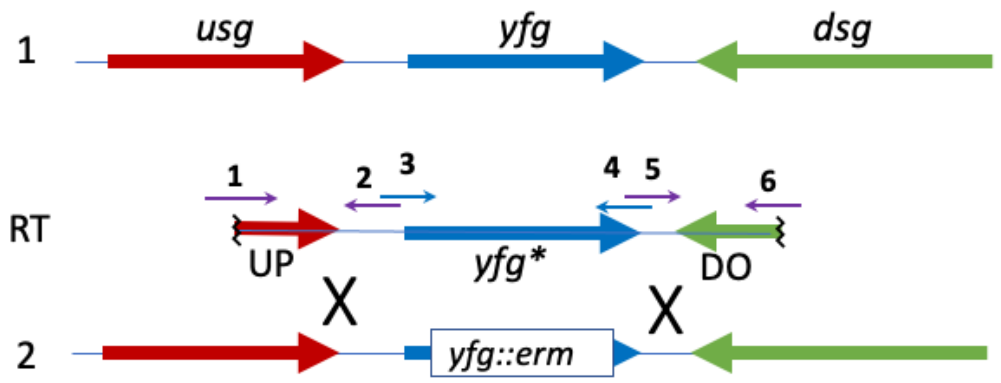

Fig. S2. **General strategy for assembly of repair template.** A generic chromosome region (1) is shown, containing your favorite gene (*yfg*) flanked by an upstream gene (*usg*) and a downstream gene (*dsg*). To alter *yfg* (or transfer a different *yfg* allele) into your strain, first introduce the relevant *erm* disruptant (*yfg::erm*) from the BKE collection (2). One can then introduce into strain 2 any genomic DNA containing a desired *yfg** allele by cotransformation with pAJS23 (which will cleave the *erm* cassette). Alternatively, mutations and gene fusions can be constructed using a repair template (RT) constructed by PCR-based methods. The desired cargo fragment (blue primers 3 and 4) can contain altered function alleles, gene fusions, or orthologous genes, for example. This fragment is then linked by SOEing PCR (taking advantage of complementary sequences between divergent primers 2/3 and 4/5, as shown) with an UP (primer 1 and 2) and a DO (primer 5 and 6) fragment for homologous recombination. The UP and DO fragments should be at least ~500 bp for the best efficiency. Note that to clone the RT into pAJS23, primers 1 and 6 should additionally include distinct SfiI recognition sequences. For example, primer 1 can include the 5'-sequence AAGGCCAACGAGGCC and primer 6 can include the 5'-sequence AAGGCCTTATTGGCC (see Table S2 for examples highlighted in red and blue, respectively).

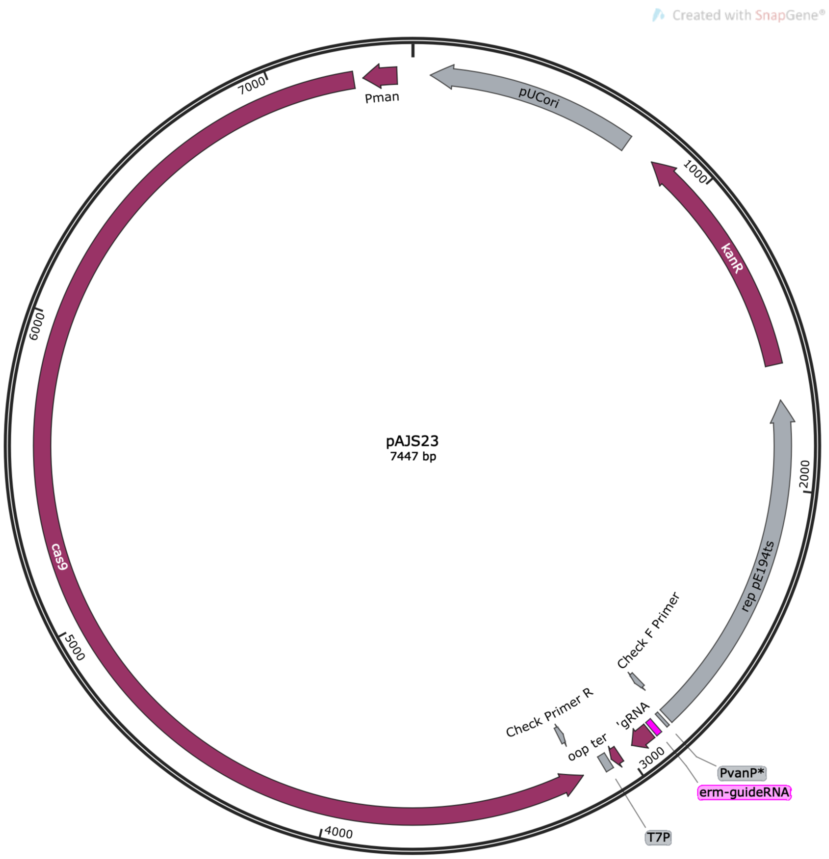

>pAJS23
gaatggccatgaccaaaatcccttaacgtgagttttcgttccactgagcgtcagaccccgtagaaaagatcaaaggatcttcttgagatcctttttttctgcgcgtaatctgctgcttgcaaacaaaaaaaccaccg
ctaccagcggtggtttgtttgccggatcaagagctaccaactctttttccgaaggtaactggcttcagcagagcgcagataccaaatactgtccttctagtgtagccgtagttaggccaccacttcaagaactctgta
gcaccgcctacatacctcgctctgctaatcctgttaccagtggctgctgccagtggcgataagtcgtgtcttaccgggttggactcaagacgatagttaccggataaggcgcagcggtcgggctgaacggggggtt
cgtgcacacagcccagcttggagcgaacgacctacaccgaactgagatacctacagcgtgagctatgagaaagcgccacgcttcccgaagggagaaaggcggacaggtatccggtaagcggcagggtcgga
acaggagagcgcacgagggagcttccagggggaaacgcctggtatctttatagtcctgtcgggtttcgccacctctgacttgagcgtcgatttttgtgatgctcgtcaggggggcggagcctatggaaaaacgcc
agcaacgcggcctttttacggttcctggccttttgctggccttttgctcacatgttctttcctgcgttatcccctgattctgtggataaccgtattaccgcctttgagtgagctgaaattatgaggggatctctcagagct
cgaggtcatcgttcaaaatggtatgcgttttgacacatccactatatatccgtgtcgttctgtccactcctgaatcccattccagaaattctctagcgattccagaagtttctcagagtcggaaagttgaccagacatt
acgaactggcacagatggtcataacctgaaggaagatctgattgcttaactgcttcagttaagaccgaagcgctcgtcgtataacagatgcgatgatgcagaccaatcaacatggcacctgccattgctacctgc
acagtcaaggatggtagaaatgttgtcggtccttgcacacgaatattacgccatttgcctgcatattcaaacagctcttctacgataagggcacaaatcgcatcgtggaacgtttgggcttctaccgatttagcagtt
tgatacactttctctaagtatccacctgaatcataaatcggcaaaatagagaaaaattgaccatgtgtaagcggccaatctgattccacctgagatgcataatctagtagaatctcttcgctatcaaaattcacttc
caccttccactcaccggttgtccattcatggctgaactctgcttcctctgttgacatgacacacatcatctcaatatccgaatagggcccatcagtctgacgaccaagagagccataaacaccaatagccttaacat
catccccatatttatccaatattcgttccttaatttcatgaacaatcttcattctttcttctctagtcattattattggtccattcactattctcattccctttttcagataattttagatttgcttttctaaataagaatatttgg
agagcaccgttcttattcagctattaaacccattatatcgggtttttgaggggatttcaactgcagacacctaaattcaaaatctatcggtcagatttataccgatttgattttatatattcttgaataacatacgccga
gttatcacataaaagcgggaaccaatcatcaaatttaaacttcattgcataatccattaaactcttaaattctacgattccttgttcatcaataaactcaatcatttctttaattaatttatatctatctgttgttgttttc
tttaataattcatcaacatctacaccgccataaactatcatatcttctttttgatatttaaatttattaggatcgtccatgtgaagcatatatctcacaagacctttcacacttcctgcaatctgcggaatagtcgcattc
aattcttctgttaattatttttatctgttcataagatttattaccctcatacatcactagaatatgataatgctctttttttcatcctaccttctgtatcagtatccctatcatgtaatggagacactacaaattgaatgtgta
actcttttaaatactctaaccactcggcttttgctgattctggatataaaacaaatgtccaattacgtcctcttgaatttttcttgttttcagtttcttttattacattttcgctcatgatataataacggtgctaatacattt
aacaaaatttagtcatagataggcagcatgccagtgctgtctatcttttttttgtttaaaatgcaccgtattcctcctttgcatattttttttattagaataccggttgcatctgatttgctaatattatatttttctttgattct
atttaatatctcattttcttctgttgtaagtcttaaagtaacagcaactttttttctcttcttttctatctacaaccatcactgtacctcccaacatctgtttttttcactttaacataaaaaacaacctttttaacattaaaaa
cccaatatttatttatttgtttggacaatggacaatggacacctagggggggaggtcgtagtaccccccctatgttttctcccctaaataaccccaaaaatctaagaaaaaaagacctcaaaaaggtctttaattaaca
tctcaaatttcgcatttattccaatttcctttttgcgtgtgatgcgaattcttgaccgtgattagagaattgagtaaaatgtacctacgTTTGAAATCGGCTCAGGAAAgttttagagctagaaatagcaagt
taaaataaggctagtccgttatcaacttgaaaaagtggcaccgagtcggtgctttttactccatctggatttgttcagaacgctcggttgccgccgggcgtttttttatctaaagcttaggcccagtcgaaagactggg
cctttttaatacgactcactatagggtcgacggccaacgaggcccgggccaataaggcctttctagattaagaaataatcttcatctaaaatatacttcagtcacctcctagctgactcaaatcaatgcgtgtttcat
aaagaccagtgatggattgatggataagagtggcatctaaaacttcttttgtagacgtatatcgtttacgatcaattgttgtatcaaaatatttaaaagcagcgggagctccaagattcgtcaacgtaaataaatg
aataatattttctgcttgttcacgtattggtttgtctctatgtttgttatatgcactaagaactttatctaaattggcatctgctaaaataacacgcttagaaaattcactgatttgctcaataatctcatctaaataatgc
ttatgctgctccacaaacaattgtttttgttcgttatcttctggactacccttcaacttttcataatgactagctaaatataaaaaattcacatatttgcttggcagagccagctcatttcctttttgtaattctccggcac
tagccagcatccgtttacgaccgttttctaactcaaaaagactatatttaggtagtttaatgattaagtctttttaacttccttatatcctttagcttctaaaaagtcaatcggatttttttcaaaggaacttctttccat
aattgtgatccctagtaactctttaacggattttaacttcttcgatttcccttttttccaccttagcaaccactaggactgaataagctaccgttggactatcaaaaccaccatattttttttggatcccagtctttttttacga
gcaataagcttgtccgaatttctttttggtaaaattgactccttggagaatccgcctgtctgtacttctgttttcttgacaatattgacttggggcatggacaatactttgcgcactgtggcaaaatctcgccctttatcc
cagacaatttctccagtttccccattagtttcgattagagggcgtttgcgaatctctccatttgcaagtgtaatttctgttttgaagaagttcatgatattagagtaaaagaaatattttgcggttgctttgcctatttctt
gctcagacttagcaatcattttacgaacatcataaactttataatcaccatagacaaactccgattcaagttttggatatttcttaatcaaagcagttccaacgacggcatttagatacgcatcatgggcatgatggt

aattgttaatctcacgtactttatagaattggaaatcttttcggaagtcagaaactaatttagattttaaggtaatcactttaacctctcgaataagtttatcattttcatcgtatttagtattcatgcgactatccaaaa
tttgtgccacatgcttagtgatttggcgagtttcaaccaattggcgtttgataaaaccagctttatcaagttcactcaaacctccacgttcagctttcgttaaattatcaaacttacgttgagtgattaacttggcgttt
agaagttgtctccaatagtttttcatctttttgactacttcttcacttggaacgttatccgatttaccacgatttttatcagaacgcgttaagaccttattgtctattgaatcgtctttaaggaaactttgtggaacaatgt
gatcgacatcataatcacttaaacgattaatatctaattcttggtccacatacatgtctcttccattttggagataatagagatagagcttttcattttgcaattgagtattttcaacaggatgctctttaagaatctga
cttcctaattctttgataccttcttcgattcgtttcatacgctctcgcgaatttttctggcccttttgagttgtctgattttcacgtgccatttcaataacgatattttctggcttatgccgccccattactttgaccaattcat
caacaacttttacagtctgtaaaataccttttttaatagcagggctaccagctaaatttgcaatatgttcatgtaaactatcgccttgtccagacacttgtgctttttgaatgtcttctttaaatgtcaaactatcatcat
ggatcagctgcataaaattgcgattggcaaaaccatctgatttcaaaaaatctaatattgttttgccagattgcttatccctaataccattaatcaattttcgagacaaacgtccccaaccagtataacggcgacgtt
taagctgtttcatcaccttatcatcaaagaggtgagcatatgttttaagtctttcctcaatcatctccctatcttcaaataaggtcaatgttaaaacaatatcctctaagatatcttcattttcttcattatccaaaaaat
ctttatctttaataatttttagcaaatcatggtaggtacctaatgaagcattaaatctatcttcaactcctgaaatttcaacactatcaaaacattctattttttttgaaataatcttcttttaattgcttaacggttactttt
cgatttgttttgaagagtaaatcaacaatggctttcttctgttcacctgaaagaaatgctggttttcgcattccttcagtaacatatttgacctttgtcaattcgttataaaccgtaaaatactcataaagcaaactatg
ttttggtagtactttttcatttggaagatttttatcaaagtttgtcatgcgttcaataaatgattgagctgaagcacctttatcgacaacttcttcaaaattccatggggtaattgtttcttcagacttccgagtcatccat
gcaaaacgactattgccacgcgccaatggaccaacataataaggaattcgaaaagtcaagatttttttcaatcttctcacgattgtcttttaaaaatggataaaagtcttcttgtcttctcaaaatagcatgcagctc
acccaagtgaatttgatggggaatagagccgttgtcaaaggtccgttgcttgcgcagcaaatcttcacgatttagtttcaccaataattcctcagtaccatccatttttttctaaaattggtttgataaatttataaaatt
cttcttggctagctcccccatcaatataacctgcatatccgtttttttgattgatcaaaaaagatttctttatacttttctggaagttgttgtcgaactaaagcttttaaaagagtcaagtcttgatgatgttcatcgtagc
gtttaatcattgaagctgataggggagccttagttatttcagtatttactcttaggatatctgaaagtaaaatagcatctgataaattcttagctgccaaaaacaaatcagcatattgatctccaatttgcgccaata
aattatctaaatcatcatcgtaagtatcttttgaaagctgtaatttagcatcttctgccaaatcaaaatttgatttaaaattaggggtcaaacccaatgacaaagcaatgagattcccaaataagccatttttcttctc
accggggagctgagcaatgagattttctaatcgtcttgatttactcaatcgtgcagaaagaatcgctttagcatctactccacttgcgttaatagggttttcttcaaataattgattgtaggtttgtaccaactggata
aatagtttgtccacatcactattatcaggatttaaatctccctcaatcaaaaaatgaccacgaaacttaatcatatgcgctaaggccaaatagattaagcgcaaatccgctttatcagtagaatctaccaattttttt
cgcagatgatagatagttggatatttctcatgataagcaacttcatctactatatttccaaaaataggatgacgttcatgcttcttgtcttcttccaccaaaaaaagactcttcaagtcgatgaaagaaactatcatct
actttcgccatctcatttgaaaaaatctcctgtagataacaaatacgattcttccgacgtgtataccttctacgagctgtccgtttgagacgagtcgcttccgctgtctctccactgtcaaataaaagagccccctataa
gatttttttgatactgtggcggtctgtatttcccagaaccttgaactttttagacggaaccttataatcatcagtgatcaccgcccatccgacgctatttgtgccgatatctaagcctattgagtatttcttatccatttt
tgcctcctaacttaagaataagatcttgtctcaactgtataccgaaatcagctcattaaaatcgcttttttttaccataggttccggtaataaaggcatttttccctataacaaaaaaagcaaggaataatccctgcttt
taataatccaaatgagataaaaatgtcatgacattggtgtacagaa

Fig. S3. **Plasmid map and DNA sequence of plasmid pAJS23.** A plasmid map of the 7447 bp pAJS23 plasmid (derived from pJOE8999, ref. 1) is shown illustrating key features. The text file contains the complete plasmid sequence, with the sequence corresponding to the anti-*erm* gRNA in uppercase.

Bibliography and References Cited

1. Altenbuchner J. 2016. Editing of the *Bacillus subtilis* Genome by the CRISPR-Cas9 System. Appl Environ Microbiol 82:5421-7.
